# A review of molecularly targeted therapy in biliary tract carcinoma: what is the next step?

**DOI:** 10.37349/etat.2021.00056

**Published:** 2021-10-31

**Authors:** Giacomo Aimar, Chiara Paratore, Clizia Zichi, Donatella Marino, Elisa Sperti, Andrea Caglio, Teresa Gamba, Francesca De Vita, Massimo Di Maio

**Affiliations:** Department of Oncology, University of Turin, Division of Medical Oncology, Ordine Mauriziano Hospital, Via Magellano 1, 10128 Turino, Italy; Humanitas University, Humanitas Research Hospital, Italy

**Keywords:** Biliary tract carcinomas, target therapy, molecular alterations, isocitrate dehydrogenase, fibroblast growth factor receptor, human epidermal growth factor receptor 2, multitarget therapy

## Abstract

Patients with unresectable biliary tract carcinomas (BTCs) have a poor prognosis with a median overall survival of fewer than 12 months following systemic chemotherapy. In recent years, the identification of distinct molecular alterations with corresponding targeted therapies is modifying this therapeutic algorithm. The aim of this review is to present an overview of targeted therapy for BTCs, describing published available data and potential future challenges in ongoing trials. From clinicaltrials.gov online database all ongoing trials for BTCs (any stage) was examinated in July 2021, and data regarding study design, disease characteristics and type of treatments were registered. Oncogenic-driven therapy (targeted therapy) was investigated in 67 trials. According to research, 15 ongoing trials (22.4%) are investigating fibroblast growth factor (FGF) receptor (FGFR)-inhibitors in BTCs. Three (18.7%) are open-label randomized multicenter phase 3 trials, 8 (50%) are single-arm phase two trials, and 4 (25%) are phase one studies. Twelve (17.9%) clinical trials dealt with isocitrate dehydrogenase (IDH) 1/2 targeting therapy either in combination with cisplatin (Cis) and gemcitabine (Gem) as first-line treatment for BTCs or in monotherapy in patients with IDH1 mutant advanced malignancies, including cholangiocarcinoma (CCA). Nine (13.4%) clinical trials tested human epidermal growth factor receptor (HER) 2 targeting therapy. Four (44.4%) studies are phase I trials, two (22.2%) are phase I/II trials, and three (33.3%) phase II trials. Rare molecular alterations in BTCs, such as anaplastic lymphoma kinase (ALK), c-ros oncogene1 receptor tyrosine kinase (ROS1), and v-RAF murine sarcoma viral oncogene homologue B1 (BRAF), are also under investigation in a few trials. Forty-four clinical trials (17.2%) are investigating not oncogenic-driven multitarget therapy like multireceptor tyrosin kinase inhibitors and antiangiogenetic agents. In conclusion, this review shows that BTCs management is experiencing important innovations, especially in biomarker-based patient selection and in the new emerging therapeutic approach. Many ongoing trials could answer questions regarding the role of molecular inhibitors leading to new therapeutic frontiers for molecular subcategories of BTCs.

## Introduction

Biliary tract carcinomas (BTCs) constitute heterogeneous diseases, arising from biliary epithelium, sub-classified in intrahepatic cholangiocarcinoma (CCA, iCCA), perihilar CCA (pCCA), extrahepatic CCA (eCCA), and gallbladder carcinoma (GBC). Potentially curative resection is amenable only in 10–15% of BTCs, with a five-year overall survival (OS) rate of 30% due to the high recurrence rate (50–60%) [1, 2]. Unresectable BTCs have a poor prognosis with a median OS of fewer than 12 months following systemic chemotherapy [3]. A combination of cisplatin (Cis) 25 mg/m^2^ and gemcitabine (Gem) 1,000 mg/m^2^ each on day 1 and 8 every 3 weeks for eight cycles is the standard first-line in BTCs, as emerged from the phase 3 ABC-02 trial, where this combination improved OS compared to Gem monotherapy [median OS 11.7 months *vs*. 8.1 months, hazard ratio (HR) = 0.64; 95% confidence interval (CI): 0.52–0.80; *P* < 0.001] [3]. Second-line treatment yields a limited outcome. The recently published phase 3 ABC-06 trial established 5-fluorouracil + oxaliplatin (FOLFOX6) as the new second-line standard treatment with an advantage in OS [median 6.2 *vs*. 5.3 months, HR = 0.69; 95% CI: 0.50–0.97; *P* = 0.031) compared with best supportive care [4].

In recent years, the identification of distinct molecular alterations that are “actionable” (with available corresponding targeted therapies) is modifying this therapeutic algorithm. The aim of this review is to present an overview of targeted therapies for BTCs, describing published available data and potential future challenges in ongoing trials. We conducted research on clinicaltrials.gov of ongoing trials for BTCs at any stage in January 2021. Studies enrolling patients with iCCA, pCCA, eCCA, and GBC at any stage were included. We gathered data regarding study design, disease characteristics, and type of treatments.

## Carcinogenesis pathways in BTCs

A marked heterogeneity characterizes BTC, not only because of intratumoral diversity due to anatomic site, ethnicity, concomitant flogosis, or viral hepatitis but also for several, often overlapping, signaling pathways involved. Among the environmental and pathologic conditions that can all promote neoplastic transformation, primary sclerosing cholangitis, cirrhosis, hepato/chole/choledocholithiasis, chronic cholecystitis, chronic non-alcoholic liver disease, hepatic C virus infection, and liver flukes infestation are the most acknowledged.

In the last decade, several research groups have proposed their own molecular classification based on high throughput techniques such as whole exome or genome sequencing, DNA methylation profiling, targeted sequencing, or single nucleotide polymorphism (SNP) array [5]. The description of each of these analyses goes beyond the purposes of this manuscript, but we can affirm that BTC generally lacks a predominant oncogenic pathway. Driver genes mutations analysis reveals that tumor protein p53 (*TP53*), Kirsten rat sarcoma 2 viral oncogene homolog (*KRAS*), mothers against decapentaplegic homolog family member 4 (*SMAD4*), neurofibromatosis type 1 (*NF1*), AT-rich interacting domain-containing protein 1A gene (*ARID1A*) are the most commonly altered. When taking into account the cohorts of iCCA, isocitrate dehydrogenase (IDH) 1 mutations, and fibroblast growth factor receptor (FGFR) translocations are pointed out as frequent, druggable alterations, as we will further discuss. Moreover, although the grouping in clusters varies a lot, it is possible to detect groups of tumors with activation of inflammation/immune pathway [interleukin (IL) 4, IL10, phosphates signal transducer and activator of transcription 3 (pSTAT3) overexpression, upregulation of programmed cell death protein 1 (PD-1), PD-1 ligand 2 (PD-L2), and B- and T-lymphocyte attenuator (BTLA)], and other groups that show a proliferation phenotype [enrichment of epidermal growth factor receptor (EGFR), rat sarcoma (RAS), mitogen-activated protein kinases (MAPK), overexpression of casein kinase II A1 (CSNK2A1), myelocytomatosis oncogene (MYC) targets, activation of cell cycle signaling and DNA repair pathways].

Currently, these classifications are speculative and do not have a clear role in routine CCA patients’ management, although, as we will further discuss, a molecular characterization may be a useful tool to allocate patients to a specific treatment in a clinically appropriate timeframe.

## Challenges for design and conduction of clinical trials in BTC

Traditionally, clinical development of new drugs included the identification of safe dose within phase I trials (often not limited to a specific type of tumor), the description of activity within phase II trials dedicated to specific tumors, the demonstration of efficacy within phase III trials comparing the experimental treatment with the best standard of care. This research paradigm has been deeply challenged in recent years, particularly for targeted agents, where randomization is considered often not feasible, both for the loss of equipoise (if early trials demonstrate strong activity, indirectly much better than the available standard) and the rarity of the disease. For both these reasons, BTC is experiencing a large evolution in the methodology of clinical trials. Especially when the activity is shown in phase I phase II trials will be particularly high, we can expect that some treatments could be authorized without the conduction of randomized phase III trials. However, if this can be considered absolutely reasonable when the experimental treatment is associated with a high probability of durable responses and disease control, in many situations, with less exceptional results, randomization would be important to quantify the incremental benefit. Many of the molecular alterations discussed in this review are present in a small percentage of an uncommon type of tumor, and this implies that, not only for industry-sponsored trials but also for academic research, participation of many centers is essential to timely reach the accrual.

Another important point to be considered is that, when a trial is designed to test a single experimental treatment for a single molecular alteration, the vast majority of screened patients will not be eligible due to a negative molecular screening. This strongly reduces the efficiency of all study procedures, including the availability of tumor tissue, reducing also the motivation of patients to participate in clinical trials. From this point of view, particularly for rare cancers like BTC, the design of “umbrella” trials, testing several targeted agents according to different alterations revealed by the molecular screening, is able to increase the efficiency of the trial, offering a promising experimental treatment for a higher proportion of subjects. Unfortunately, as we will show in this review, the majority of ongoing trials are still testing a single experimental treatment for a single molecular target.

## Discussion

**Figure 1.**
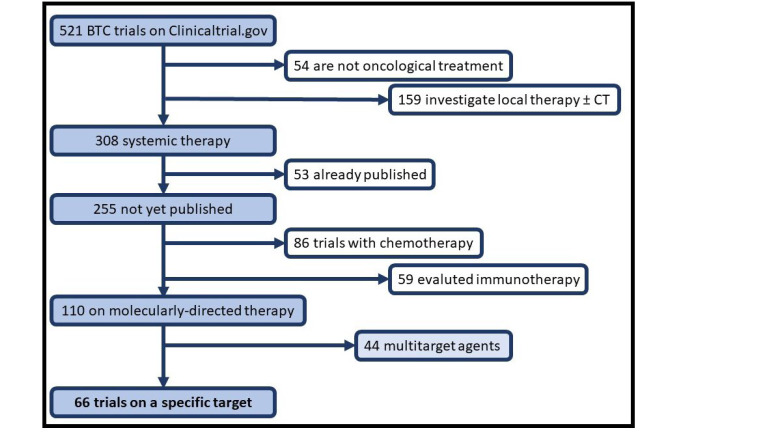
Flowchart from trials available on clinicaltrials.gov. CT: computed tomography

In 2021, updated in July, 522 ongoing trials of BTCs were available in clinicaltrials.gov: 54 are not oncological treatment, 159 of local therapy (radiotherapy, surgery, or interventional radiology alone or associated with chemotherapy), and 309 of systemic treatment [chemotherapy, immunotherapy (IT), targeted therapy and other], 53 trials were previously published and not included in our analysis. Among these, oncogenic driven therapy (targeted therapy) was investigated in 67 trials, and multitarget therapy in further 44 trials (Figure 1). Depending on the type of targeted assessment, studies were divided into: oncogene-driven studies (molecular alteration was reported as an inclusion criterion) or non-oncogene-driven studies, where genetic mutation was considered for outcome assessment and response to treatment. Trials with non-oncology outcomes or treatment were excluded, such as trials with anesthetics tested during endoscopic retrograde cholangiopancreatography or with enteral nutrition after surgery. Molecular alterations were evaluated in clinical trials with complementary techniques: immunohistochemistry (IHC), fluorescence in situ hybridization (FISH), or next generation sequencing (NGS).

In the past decade, some attempts of integrating targeted therapies into the treatment of BTC have been made. In many cases, targeted agents have been tested according to a “one size fits all” approach, without molecular selection according to putative biomarkers and predictive factors. Many agents, such as anti-EGFR cetuximab or panitumumab, or anti-vascular endothelial growth factor (VEGF) bevacizumab, have failed to improve the outcomes [6–9]. In our review, we will focus on the ongoing trials, with the aim of pointing out the most promising pathways.

### Targeting FGFR-family

The FGFR pathway is involved in many physiological cellular processes and cancer cells’ proliferation, migration, and survival [10]. The FGFR family consists of four transmembrane receptors, FGFR1-4. Single nucleotide variants, gene fusions, and copy number amplifications were found by NGS analysis, reporting aberrant activation of this signal in 5–10% of human cancers [11]. This percentage increases up to an average of 15% in BTC, mostly in iCCA [12]. Fusions or rearrangements of FGFR2 are the most frequent alteration, which is mutually exclusive with other well-known mutations [KRAS, v-RAF murine sarcoma viral oncogene homologue B1 (BRAF), or IDH1] [13]. Given the poor prognosis of BTCs, targeting this pathway is an attractive therapeutic goal.

Pemigatinib is the first Food and Drug Administration (FDA)-approved drug in advanced previously treated FGFR-altered BTC. Pemigatinib is a selective, reversible, oral inhibitor of FGFR1, 2, and 3. In the phase II Fight-202 study, 35% of patients achieved an objective response (95% CI: 26.5–45.4), with no safety warning or treatment-related deaths [14]. Infigratinib (an irreversible pan-FGFR inhibitor) was recently approved by FDA for previously treated advanced BTC harboring an FGFR2 fusion or rearrangement. In a phase II study, 108 patients were enrolled (77% with *FGFR2* fusions), achieving an objective response rate (ORR) of 23.1% in the intention-to-treat population, 34% in the second-line, and 13.8% in later lines [15]. In LUC2001, the selective inhibitor of pan-FGFR erdafitinib was tested on 14 Asian patients with previously treated FGFR altered CCA. Erdafitinib achieved an ORR of 50% and a median duration of response (DoR) of 6.83 months [60% and 100% of ORR and disease control rate (DCR) respectively in FGFR2^+^ restricted patients] [16]. Derazantinib, a multi-kinase inhibitor with potent pan-FGFR activity, was tested on 29 iCCAs with FGFR2 fusions, reporting an ORR of 20.7% and a DCR of 82.8% [17]. The multicenter ongoing phase II FOENIX-CCA2 study is testing futibatinib (an irreversible inhibitor) in BTC with any alteration of the FGFR family. The planned accrual is over 800 patients. In the interim analysis, an ORR of 37.3%, a DCR of 82.1%, and a median DoR of 8.3 months were reported [18].

According to our search, 16 ongoing trials (25%) are investigating FGFR-inhibitors in BTCs (as described in Table 1). Three (18.7%) are open-label randomized multicenter phase III trials, 8 (50%) are single-arm phase II trials and 4 (25%) are phases I studies. A further study, testing the association of anti-FGFR and anti-IDH treatment, will be discussed in the section dedicated to IDH (NCT04088188).

**Table 1.** Active trials, both recruiting and not recruiting, with FGFR’s inhibitors described above

**NCT**	**Phase**	**Status**	**Tumors**	**Line of treatment**	**Target**	**Experimental treatment**	**Standard treatment**	**Primary endpoints**	**Sponsor**
NCT03656536	3	Recruiting	CCA	1 L	FGFR2 rearrangement	Pemigatinib	Gem-Cis	PFS	Profit
NCT03773302	3	Recruiting	CCA	1 L	FGFR2 fusions/translocations	Infigratinib	Gem-Cis	PFS	Profit
NCT04093362	3	Recruiting	iCCA	1 L	FGFR2 rearrangement	Futibatinib	Gem-Cis	PFS	Profit
NCT04256980	2	Active, not recruiting	CCA	2 L	FGFR2 rearrangement	Pemigatinib	N.A.	ORR	Profit
NCT04083976	2	Recruiting	CCA	≥ 2 L	FGFR mutations/fusions	Erdafitinib	N.A.	ORR	Profit
NCT03230318	2	Recruiting	iCCA	2 L	FGFR2 fusion, mutation or amplification	Derazantinib	N.A.	ORR	Profit
NCT04233567	2	Recruiting	CCA	≥ 2 L	FGFR fusions, translocations or activating mutations	Infigratinib	N.A.	ORR	Non-profit
NCT04238715	2	Recruiting	CCA	2 L	FGFR2 fusion	E7090	N.A.	ORR	Profit
NCT04353375	2	Not yet recruiting	iCCA	2 L	FGFR2 fusion	HMPL-453 tartrate	N.A.	ORR	Profit
NCT04565275	1/2	Recruiting	CCA	NTA	FGFR alteration	ICP-192	N.A.	MTD, ORR, AE, OBD, RP2D	Profit
NCT03144661	1	Terminated	CCA	NTA	FGF19/FGFR4	INCB062079	N.A.	Safety	Profit
NCT04149691	1	Recruiting	CCA	NTA	FGFR 1, 2, 3 alteration	CPL304110	N.A.	MTD, safety	Profit
NCT03583125	1	Recruiting	CCA	NTA	FGFR alteration	EOC317	N.A.	DLT	Profit
NCT04526106	1	Recruiting	CCA	NTA	FGFR2 fusion, mutation, or amplification	RLY-4008	N.A.	MTD, AE	Profit

A “withdrawn” trial was excluded, stopped early because of a slow accrual (NCT04479904). AE: adverse event; DLT: dose-limiting toxicity; MTD: maximum tolerated dose; NTA: no treatment available; OBD: optimal biological dose; PFS: progression-free survival; RP2D: recommended phase 2 dose; L: chemotherapy line; N.A.: not applicable

Three are phase III trials testing FGFR-inhibitors *versus* Gem-Cis doublet in the first line. These trials involve untreated FGFR2 rearranged BTC, with PFS as the primary endpoint. FIGHT-302 trial is an active-controlled study that enrolls patients with BTCs in Europe and the United States, testing the efficacy of pemigatinib. Accrual is expected to be completed in 2026 (NCT03656536). FOENIX-CCA3 is a trial that aims to evaluate futibatinib only in patients with iCCA. Results are awaited in 2022 (NCT04093362). PROOF 301 trial randomizes patients with advanced CCA with *FGFR2* gene fusions/translocations to receive infigratinib *versus* standard therapy. Data are expected in 2024 (NCT03773302). The results of these trials could potentially define a new standard targeted therapy in the front-line setting for molecularly selected advanced BTC.

Furthermore, there is a huge number of phase II studies with a more complex landscape. All these studies enroll patients after progression to first-line Gem-platinum-based chemotherapy, have a single-arm design and ORR is the primary endpoint. A phase II study with pemigatinib is ongoing in China, in BTC characterized by FGFR2 rearrangement, to evaluate its efficacy in the Eastern population (NCT04256980). Erdafitinib and derazantinib are investigated also in phase II trials with a different design from the studies mentioned above. Notably in erdafitinib study, BTC is included if FGFR fusions or mutations are present (NCT04083976). FIDES-01 with derazantinib enrolls the only iCCA with FGFR amplification, mutations, and fusions (NCT03230318). The basket trial Foenix 101 consists of three parts, including a phase II to evaluate infigratinib in iCCA harboring *FGFR2* gene fusions. Interestingly, in one cohort, infigratinib has been tested in patients previously treated with FGFR inhibitor (NCT04233567). Finally, 3 further phase II studies, similar in design and endpoint, are ongoing. The drugs tested are HMPL-453, E7090, and ICP-192. The first enroll only iCCA (NCT04353375; NCT04238715; NCT04565275). Another phase II trial with famitinib has been withdrawn for slow accrual (NCT04479904).

Four studies are phase I basket trials enrolling previously treated BTC with safety as the primary endpoint (NCT03144661; NCT04149691; NCT03583125; NCT04526106).

In our opinion, although FGFR alterations affect only a small percentage of iCCA, targeting this pathway could be a valuable option. In particular, data from the FIGHT-302 trial are strongly awaited because, if positive, we could spare our patients systemic chemotherapy in favor of a tailored and less toxic treatment. In addition, we reckon that an effort should be made to early detect the mechanisms of resistance to FGFR inhibitors as a proactive research strategy. Likewise, in a phase II trial of infigratinib, a cohort of FGFR-treated patients was included.

### Targeting IDH & breast cancer gene

The IDH proteins are critical metabolic enzymes, members of the oxidoreductase family. Cancer-associated mutations have been identified in two of the three existing isoforms: IDH1 and IDH2, which are mutually exclusive. They occur in approximately 10–12% of iCCA, while their incidence in eCCA and GBC is rare [19]. Almost all IDH mutations are missense, confined to a single residue (R132) for IDH1, in the active site of the enzyme [20]. These mutations play a role in the pathogenesis of CCA by blocking hepatocyte differentiation and promoting cellular proliferation [21].

Most IDH inhibitors target isoform 1. The first-in-class IDH1 inhibitor is ivosidenib (AG-120), an oral small molecule, already approved by FDA for patients with IDH-mutant acute myeloid leukemia. In the multicenter, randomized, double-blind, placebo-controlled phase III ClarIDHy study, ivosidenib significantly improved PFS (2.7 *vs*. 1.4 months, HR = 0.37; 95% CI: 0.25–0.54; one-sided *P* < 0.0001) in IDH1-mutant, chemo-refractory patients [22]. Researchers presented final OS data at the 2021 Gastrointestinal Cancers Symposium, showing a difference that becomes statistically significant after adjusting for crossover (HR = 0.49, *P* < 0.0001) [23].

We found nine (14.1%) clinical trials with IDH 1/2 targeting therapy (see Table 2). Ivosidenib, in combination with Cis and Gem, is currently investigated in a multicenter phase I trial (NCT04088188) in treatment-naive patients. The primary objective is to evaluate safety, tolerability, MTD, and RP2D.

**Table 2.** Active trials, both recruiting and not recruiting, testing IDH and breast cancer gene (BRCA) inhibitors

**NCT**	**Phase**	**Status**	**Tumors**	**Line of treatment**	**Target**	**Experimental treatment**	**Standard treatment**	**Primary endpoints**	**Sponsor**
NCT04088188	1	Recruiting	CCA	1 L	IDH1	Ivosidenib + Cis-Gem	N.A.	DLT	Profit
NCT04521686	1	Recruiting	CCA	NTA	IDH1	LY3410738	N.A.	RP2D	Profit
NCT02381886	1	Active, not recruiting	CCA	UNK	IDH1	IDH305	N.A.	DLT	Profit
NCT03684811	1/2	Active, not recruiting	CCA	Phase 1: NTA Phase 2: 1	IDH1	Olutasidenib +/– Nivolumab or +/– Cis-Gem	N.A.	DLT, RP2D, ORR	Profit
NCT02273739	1/2	Completed	iCCA	NTA	IDH2	Enasidenib	N.A.	AEs, DLT	Profit
NCT02496741	1/2	Completed	iCCA	All	IDH1/2	Metformin + chloroquine	N.A.	MTD	No-profit
NCT03878095	2	Recruiting	CCA	NTA	IDH1/2 + PARP*	Ceralasertib + olaparib	N.A.	ORR	No-profit
NCT03212274	2	Recruiting	CCA	NTA	IDH 1/2	Olaparib	N.A.	ORR	No-profit
NCT03991832	2	Recruiting	CCA	1–2 L	IDH	Olaparib + durvalumab	N.A.	ORR, DCR	No-profit
NCT03207347	2	Recruiting	CCA	NTA	DDR mutation	Niraparib	N.A.	ORR	Profit
NCT04306367	2	Recruiting	CCA	2 L	PARP*	Pembrolizumab + olaparib	N.A.	ORR	No-profit
NCT01282333	1	Terminated	eCCA and gallbladder cancer	1 L	BRCA1/2	Veliparib + Cis-Gem	N.A.	MTD	No-profit

We also reported two trials defined as “completed” and another one defined as “terminated” on www.clinicaltrials.gov, whose results have never been published.

* Not inclusion criteria; UNK: unknown; PARP: poly ADP-ribose polymerase; DDR: DNA damage repair

Three other promising IDH1 inhibitors, able to cross the blood-brain barrier, are currently under investigation in patients with IDH1 mutant advanced malignancies, including CCA. LY3410738 is evaluated in a multicenter, phase I trial (NCT04521686) actively recruiting patients (estimated enrollment 180), to define the RP2D. Other two trials have already completed the accrual: a phase I trial (NCT02381886), that enrolled 166 patients to estimate the MTD and RP2D of IDH305; a phase I/II trial (NCT03684811), whose primary objective is to evaluate DLT, dose recommended for future studies and ORR of olutasidenib (FT-2102). The latter study is divided into two parts, to test olutasidenib single agent and the combination with nivolumab (hepatobiliary tumors) and the combination with Cis plus Gem (iCCA).

The development of selective IDH2 inhibitors has given less favorable results. Enasidenib (AG-221) has been investigated in a phase I trial (NCT02273739), consisting of a dose-escalation phase to determine MTD followed by expansion cohorts to further evaluate safety and tolerability. However, enrollment was closed at the end of the dose-escalation phase, and the expansion phase was not conducted.

Although the ClarIDHy study was the first positive trial of targeted therapy in BTC, the clinical relevance of the results obtained with this drug is still questioned and the approval by regulatory authorities is still pending. We think that clinical research with IDH-inhibitors should not focus on monotherapy in chemo-refractory disease, but in our opinion, a more challenging setting will be their early use in combination with chemotherapy or other targeted therapies. As an example, preclinical studies showed that IDH mutations could change cell metabolism with a predominance of oxidative metabolism [24]. A phase I/II trial (NCT02496741) is testing the combination of the oral antidiabetic metformin and the oral antimalarial drug chloroquine, to specifically inhibit these metabolic processes in patients with IDH1/2 mutated iCCA. The primary endpoint is the identification of MTD of both drugs, and the trial completed the accrual, enrolling 15 participants.

DDR pathway is related to genomic instability and cancer susceptibility. Homologous recombination (HR), mismatch repair (MMR), base excision repair (BER), and other mechanisms have been associated with CCA, but correlation to actionable target and precision medicine remains still unclear. The presence of DDR alteration could also confer a different sensitivity to platinum-based chemotherapy [25, 26]. Other recent research showed elevated DNA damage in IDH-mutant CCA, and treatment strategies using a synthetic lethality approach with PARP inhibitors are in development [27]. Three phase II trials adopting this strategy are currently recruiting patients: these studies are testing olaparib alone (NCT03212274) or in combination with ceralasertib (AZD6738-NCT03878095) or durvalumab (NCT03991832) in both IDH1 and IDH2 mutant recurrent CCA. Interest in PARP inhibitors is growing, and three phase II trials (4.5%) are testing their use in patients with molecular alterations other than IDH1/2. The NCT03207347 trial is evaluating the activity of niraparib in tumors with BRCA1 associated protein-1 (BAP1) and other selected DNA damage response pathway genes. The combination of olaparib and pembrolizumab is investigated in patients who have progressed to the first-line therapy in the trial NCT04306367. We also report a phase I trial (NCT01282333) testing the combination of veliparib with Cis and Gem in treatment-naive, advanced eCCA with known or suspected BRCA1/2 germline mutation. The trial is terminated, but the results are still unpublished.

### Targeting human epidermal growth factor receptor 2

The epidermal growth factor receptor (EGFR) kinase family consists of four receptors [ErbB1/EGFR, ErbB2/human epidermal growth factor receptor (HER) 2, ErbB3, and ErbB4] that play a crucial role in cell proliferation, differentiation, and motility and may lead to cancer development. The HER2 overexpression, gene amplification, or mutation have been described in breast, gastric, lung, and colorectal cancer. HER2 is a predictive biomarker for anti-HER2 target therapies and could be tested by IHC, FISH, and NGS.

A systematic review and meta-analysis showed a HER2 expression rate of 26.5% in BTCs, with different detection rates according to the tumor site and the testing procedures [28]. Considering the studies in which HER overexpression was defined by moderate/strong IHC expression, HER2 positive rate was 20% in eCCC and 5% in iCCA. In a cohort of 1,863 patients evaluated by NGS, Jacobi et al. [29] reported HER2 alteration in 10% of eCCA and 4% of iCCA. Despite the not negligible number of HER2 molecular alterations in BTCs, only a few clinical prospective data are available in this setting.

In a pilot study, 4 patients with HER2-positive BTCs were treated with trastuzumab plus Cis and Gem. Two patients experienced a partial response (PR, 50%), and 2 obtained stable disease (SD, 50%), with a median PFS of 6.1 months. Despite the small sample size, this pilot study showed encouraging data regarding HER2-target therapy plus standard chemotherapy, without safety warning [30].

The MyPathway trial, a multi-basket phase IIA trial, investigated pertuzumab + trastuzumab in 11 patients with HER2-positive refractory advanced BTCs. Among the 7 patients with HER2 overexpression or amplification, 2 achieved PR (29 %) and 3 SD for more than 4 months (38%) [31]. According to these promising results, pertuzumab plus trastuzumab could be further explored in HER-positive BTCs.

Promising results come from other new drugs, such as the antibody-drug conjugate trastuzumab-deruxtecan [32], the anti-HER2 antibodies mergetuximab [33], and zanidatamab, the novel antibody that simultaneously binds 2 distinct epitopes of HER2 [34].

Tailored therapy could be a novel therapeutic strategy for BTCs harboring HER2 molecular alterations, but further prospective data are needed to better understand the right workflow for HER2 testing and treating.

We found 9 (14.1%) clinical trials with HER2 targeting therapy (as shown in Table 3). HER2-positivity is a required inclusion criterion in all studies, defined by the presence of HER2 protein overexpression by IHC or by the presence of *HER2* gene amplification documented with in situ hybridization (ISH) or FISH. NGS is also allowed for HER2 testing in 5 studies (55.6 %). HER2 mutation is an inclusion criterion in only one trial (11.1%). BTCs with low HER2 expression, defined by at least 1+ at IHC, could be enrolled in two trials (22.2%).

**Table 3.** Active trials, both recruiting and not recruiting, testing the HER2 pathway

**NCT**	**Phase**	**Status**	**Tumors**	**Line of treatment**	**Target**	**Experimental treatment**	**Standard treatment**	**Primary endpoints**	**Sponsor**
NCT03613168	2	Completed	CCA	1 L	HER2	Cis + Gem + trastuzumab	N.A.	ORR, AEs	No-profit
NCT04430738	1/2	Recruiting	CCA	≥ 1 L (not prior oxaliplatin)	HER2	FOLFOX or CAPOX + tucatinib + trastuzumab	N.A.	AEs	Profit
NCT03602079	1/2	Recruiting	CCA	NTA	HER2	A166 (ADC targeting HER2)	N.A.	MTD, ORR	Profit
NCT00004074	1	Completed	eCCA and GBC	NTA	HER2	IL12 + trastuzumab	N.A.	MTD	No-profit
NCT04660929	1	Recruiting	CCA	NTA	HER2	CT-0508 (anti-HER2 CAR macrophages)	N.A.	Safety, tolerability and feasibility	Profit
NCT04466891	2	Recruiting	CCA	≥ 2 L	HER2	Zanidatamab	N.A.	ORR	Profit
NCT04579380	2	Recruiting	CCA	≥ 2 L	HER2	Tucatinib + trastuzumab	N.A.	ORR	Profit
NCT03821233	1	Recruiting	CCA	NTA	HER2	ZW49	N.A.	AEs, safety	Profit
NCT00005842	1	Completed	CCA	≥ 1 L		Trastuzumab + tipirfanib	N.A.	MTD, antitumor activity	No-profit

We also reported three trials defined as “completed” on www.clinicaltrials.gov, whose results have never been published. CAPOX: capecitabine + oxaliplatin; CAR: chimeric antigen receptor; ADC: antibody-drug conjugate

Seven (77.7%) are basket trials including different malignancies and only two trials (22.2%) are specifically designed for BTCs. Four (44.4%) studies are phase I trials investigating MTD determined according to DLT and incidence of AEs. Two (22.2%) are phase I/II trials evaluating both safety and efficacy. Three (33.3%) phase II trials have response rate (RR) as the primary endpoint and other outcomes of efficacy and tolerability as secondary endpoints. All studies are non-randomized, mostly have single group assignments (66.6 %) and two (22.2%) have sequential assignments. Eight trials (88.8%) are multicentre and are available mostly in United States (88.8%). Only three (33.3%) are not sponsored by a pharmaceutical company.

Advanced BTCs, without subtypes restriction, are included in the majority of these clinical trials: restriction due to the tumor intrahepatic or extrahepatic origin is taken into account only in one study (11.1%). One study (11.1%, NCT03613168) is investigating a first-line combination (trastuzumab plus Cis-Gem). Another study (11.1%) include patients with both treatment-naive and previously treated BTCs to evaluate the combination of trastuzumab plus the farnesyltransferase inhibitor tipirfanib (NCT00005842). Patients with BTCs in further lines of therapy (≥ 2) are included in the remaining trials (77.7 %). Trastuzumab is the main targeted agent used in further line in three trials (42.8 %): plus tucatinib and oxaliplatin-based chemotherapy (NCT04430738), plus IL12 (NCT00004074), or plus tucatinib monotherapy (NCT04579380). Anti-HER2 CAR in engineered macrophages is a potential therapeutic approach in one ongoing study (14.2 %, NCT04660929). Zanidatamab is currently investigated in patients with previously treated BTCs in one phase II trial (14.2%, NCT04466891). ADC targeting HER2 positive tumors are used as an experimental treatment in two (28.5%) clinical trials (NCT03821233, NCT03602079).

So far, anti-HER2 therapies have yielded very limited evidence but promising data of the activity. A major barrier is due to the rarity of this alteration, although is relatively more common in eCCA.

Several clinical trials are currently investigating the role of anti-HER2 target therapy in BTCs but, because of the difficulties we have just discussed, the most popular researching approach involves basket studies. Whenever an alteration is rare in a rare disease, innovative study designs could help the clinicians to match the proper drug to the appropriate patient. Most studies are in early phases, but this could not exclude a breakthrough approval in case of exceptional results.

### Targeting MAPK pathway

MAPK cascades are implicated in the survival and proliferation of tumor cells; targeting these signaling cascades has proven to be effective in many types of tumor histology (for example melanoma, lung, and colon cancer) [35, 36]. Anti-EGFR monoclonal antibodies are not as effective in BTC as in other types of gastrointestinal cancer. Both cetuximab and panitumumab, even in a KRAS wild-type (wt) population did not demonstrate an OS advantage [6–8]. One study evaluated erlotinib plus cetuximab in KRAS wt selected patients but until now no data were published (NCT00397384). Conversely, the promising activity of targeting BRAF mutated BTC arise from the phase II ROAR study.

A phase I study with AMB13-10, a BRAF inhibitor, is ongoing in patients with documented BRAF V600 mutation (NCT04190628). No phase III studies are ongoing, probably due to the rarity of this mutation, which accounts for 5% of iCCA.

Finally, there is also an interesting phase II study with MAPK kinase (MEK) inhibitor (trametinib) associated with hydroxychloroquine (autophagy inhibitor) in patients with KRAS mutation (NCT04566133) (See Table 4).

**Table 4.** Not published trials, both recruiting and not recruiting, targeting MAPK pathway

**NCT**	**Phase**	**Status**	**Tumors**	**Line of treatment**	**Target**	**Experimental treatment**	**Standard treatment**	**Primary endpoints**	**Sponsor**
NCT00397384	1	Completed	eCCA	Non specified	KRAS wild type	Erlotinib + Cetuximab	N.A.	MTD	No-profit
NCT04190628	1	Recruiting	CCA	NTA	BRAF V600 mutation	ABM-1310	N.A.	MTD/RP2D	Profit
NCT04566133	1	Not yet recruiting	CCA	NTA	KRAS mutation	Trametinib + HCQ	N.A.	PFS	Profit

HCQ: hydroxychloroquine

### Targeting anaplastic lymphoma kinase and reactive oxygen species

Aberrant forms and expression of anaplastic lymphoma kinase (ALK), a receptor tyrosine kinase from the insulin receptor superfamily, are implicated in tumorigenesis. The receptor tyrosine kinase c-ros oncogene1 receptor tyrosine kinase (ROS1) belongs to the insulin receptor family and is an oncogenic driver in different malignancies.

A post-hoc analysis retrospectively evaluated the aberrant expression of ROS1, ALK, and mesenchymal to epithelial transition (MET) in 110 patients with advanced BTCs treated with Gem plus oxaliplatin (GEMOX) with or without cetuximab, using IHC. Eighteen patients (16.3%) had IHC score intensity 3+ for any markers ROS1, ALK, and c-MET (R.A.M. high), and 92 (83.6 %) had IHC score intensity lower than three for any markers (RAM low). All RAM high BTCs were iCCA (100%) with worse OS than RAM low iCCA (median 5.7 *vs*. 11.7 months, *P* = 0.021) [37].

Sporadic cases of BTCs with ALK rearrangement or alterations and ROS1 fusion are described in few case reports [38, 39] and preclinical studies [40–42].

ALK and ROS1 molecular alterations in BTCs are a neglected topic in literature, with scarce emerging data regarding incidence and targeted anticancer drugs. Riding the wave of successes of ALK and ROS1 targeted therapy in non-small cell lung cancer (NSCLC), few clinical trials are testing these treatments in ALK and ROS1 positive BTCs population.

Three non-randomized phases II trials (4.7%) have enrolled patients with ALK and ROS1 alterations in advanced BTCs, two are terminated and only one is actually ongoing. An international multicentre basket trial is evaluating the role of entrectinib in first or subsequent lines in patients with activated neurotrophic tyrosine receptor kinase (NTRK) 1/2/3, ALK, or ROS1 pathway in different malignancies (NCT02568267). The primary endpoint is ORR. Entrectinib has already been approved by FDA and European Medical Association (EMA) for the treatment of patients with solid tumors, included BTC, expressing an *NTRK* gene fusion.

### Targeting other pathways

There are other clinical studies that test targeted agents in different pathways not mentioned above, to to briefly describe them, Table 5 has been drawn up.

**Table 5.** Active trials, both recruiting and not recruiting, testing targets not aforementioned (IDH, FGFR, PARP, HER2, ALK, ROS, and the MAPK pathway)

**NCT**	**Phase**	**Status**	**Tumors**	**Line of treatment**	**Target**	**Experimental treatment**	**Standard treatment**	**Primary endpoints**	**Sponsor**
NCT04491942	1	Recruiting	CCA	UNK	ATR*	Cis + BAY 1895344 +/– Gem	N.A.	AEs, RP2D	No-profit
NCT03829436	1	Recruiting	CCA	NTA	PPAR-alfa*	TPST-1120 +/– nivolumab	N.A.	DLT, AEs, MTD	Profit
NCT04430842	1	Recruiting	CCA	NTA	LAT1	QBS10072S	N.A.	MTD	Profit
NCT04152018	1	Recruiting	eCCA	UNK	Integrin alpha-V/beta-8*	PF 06940434 +/– IT	N.A.	DLT, AEs, ORR, PFS, DOR	Profit
NCT03422679	1/2	Recruiting	CCA	NTA	NOTCH*	CB-103	N.A.	DLT, ORR	Profit
NCT03907852	1/2	Recruiting	CCA	≥ 2 L	Protein mesothelin	Gavo-cel	N.A.	3 months ORR	Profit
NCT04068194	1/2	Recruiting	CCA and gallbladder cancer	NTA	DNA activated protein kinase (DNA-PK)*	Nedisertib	N.A.	MTD, ORR	Profit
NCT03633773	1/2	Recruiting	iCCA	1 L	Glycosylated Mucin1	MUC-1 CART cell IT	N.A.	DCR	No-profit
NCT03768375	2	Recruiting	eCCA, gallbladder cancer	1 L	Precision target therapy based on tumor molecular profiling	FORFIRINOX or cetuximab or trastuzumab or gefitinib or lapatinib or everolimus or sorafenib or crizotinib	FOLFIRINOX	PFS	No-profit
NCT03801915	2	Recruiting	CCA	Perioperative	CA 19-9 epitope	MVT-5873	N.A.	1-year recurrence rates, safety	Profit
NCT04034238	2	Recruiting	eCCA	2 L	Protein mesothelin	LMB-100 + tofacitinib	N.A.	Safety, timing of anticorpal response	Profit
NCT04383210	2	Recruiting	CCA and gallbladder cancer	NTA	*NRG1* gene fusion	Seribantumab	N.A.	ORR	Profit
NCT03102320	1b	Completed	CCA	UNK	Protein mesothelin	Anetumab ravtansine + chemotherapy	N.A.	MTD, ORR, DOR	Profit
NCT00101972	1	Completed	CCA and gallbladder cancer	2–4 L	Glycotope RAAG12*	RAV12	N.A.	Toxicity by CTCAE	Profit
NCT00020579	1	Completed	CCA and gallbladder cancer	NTA	Histone deacetylase*	Entinostat	N.A.	DLT, MTD, pharmaco-kinetics	No-profit
NCT00027534	1	Completed	Gallbladder cancer	Received prior therapy with possible survival benefit or refused such therapy	CEA	TRICOM-CEA(6D)	N.A.	Safety and feasibility	No-profit
NCT02836847	2	UNK	eCCA and gallbladder cancer	1 L	Precision target therapy based on tumor molecular profiling	GEMOX + cetuximab or trastuzumab or gefitinib or lapatinib or everolimus or sorafenib or crizotinib	GEMOX	PFS	No-profit
NCT04895046	2	Not yet recruiting	CCA	Maintenance	Defined HRD signature	Niraparib and dostarlimab	N.A,	PFS	Profit
NCT04801095	1	Recruiting	CCA	1 L	pTyr-mtRTK	WM-S1-030	N.A.	MTD	Profit
NCT05001282	1/2	Not yet recruiting	CCA	NTA	FRα over-expressing	ELU001	N.A.	MTD/RP2D	Profit

We reported four trials defined as “completed” on www.clinicaltrials.gov, whose results have never been published. Were excluded a “terminate” trial, that is a study stopped early, whose participants are no longer being examined or treated (NCT00012246), and two “withdrawn” trials, stopped early, before enrolling their first participants (NCT01501604 and NCT01859182). *Not inclusion criteria; ATR: ataxia-telangiectasia mutated (ATM) and RAD3-related; LAT: large amino acid transporter; FOLFIRINOX: leucovorin calcium, fluorouracil, irinotecan hydrochloride and oxaliplatin; CA: carbohydrate antigen; NRG1: neuroregulin1; CTCAE: common terminology criteria for adverse events; CEA: carcinoembryonic antigen; HRD: homologous recombination deficency; RTK: receptor tyrosine kinases; FRα: folate receptor alpha

### Multitarget drugs and antiangiogenetic agents

Multitarget drugs can inhibit several cell signaling pathways involved in cancer development, growth, and spreading. Plenty of multitarget inhibitors, with or without chemotherapy, had been examined in advanced BTCs in different studies with conflicting results. For example, five studies failed to show a survival benefit with sorafenib, a multikinase inhibitor targeting tyrosine kinase receptor involved in tumorigenesis [CD117 and REarranged during Transfection (RET)] and angiogenesis [VEGF receptor (VEGFR) 1, 2, 3 and platelet-derived growth factor receptor (PDGFR) β] [43–47]. In a single-arm phase II study, refractory BTCs received lenvatinib 24 mg orally daily resulting in an ORR of 11.5 % and median overall survival (mOS) of 7.35 months, with a relevant rate of grade ≥ 3 AEs (80 %) [48].

Angiogenesis creates a supportive microenvironment and promotes tumor growth and metastasis. The VEGF is expressed in 54 % and 59% of iCCA and eCCA respectively [49, 50]. Several clinical trials investigated bevacizumab in combination with chemotherapy or erlotinib in untreated and pre-treated BTCs, with modest results [51–53].

Forty-five clinical trials (15.2%) are investigating not oncogenic-driven multitarget therapy (Table 5). Lenvatinib, a multireceptor tyrosine kinase inhibitor, is currently been investigated in 9 monocentre phase II clinical trials (20.9 %) in China. In eight (88.8 %) clinical trials lenvatinib is used as a systemic treatment in first or further line for locally advanced BTCs, as monotherapy, or in association with different drugs (PD-1 monoclonal antibody or chemotherapy). The remaining 36 not oncogenic driven clinical trials are evaluating EGFR tyrosine-kinase inhibitors (TKI) and VEGF TKI.

## Conclusions

The discovery of new actionable molecular alterations, together with advances in technology, is paving the way to a new diagnostic paradigm of BTCs, leading to a novel oncogenic-driven treatment landscape.

We conducted a review of all ongoing trials in BTCs in January 2021, focusing on target therapy. In order to offer a complete landscape of the clinical research ongoing in this field, all ongoing trials were included, irrespective of disease stage or therapeutic approach. In order to improve the accuracy of the search, multiple researchers were involved to avoid collecting errors.

However, our review was conducted in one single database (ClinicalTrials.gov), thus this is not comprehensive research of all BTCs trials. We are aware that many ongoing trials could have been registered on other platforms. For example, the American Society of Clinical Oncology (ASCO)’s Targeted Agent and Profiling Utilization Registry (TAPUR) is currently enrolling patients in 130 different centers, in order to evaluate the safety and efficacy of FDA-approved drugs matched to the genomic profiles of cancer, including BTCs.

Among the discussed trials, although potentially each of the tested drugs could produce a positive result deserving further development in this setting, we think that ongoing studies with FGFR inhibitors for iCCA and HER2 inhibitors for eCCA might provide potential practice-changing results.

In conclusion, our review shows that BTCs management is experiencing several important innovations, especially in biomarker-based patient selection and in new emerging therapeutic approaches. Many ongoing trials could answer questions regarding the role of molecular inhibitors leading to new therapeutic frontiers for molecular subcategories of BTCs. Given the spectrum of heterogeneity of BTCs, clinical studies designed around molecular alterations are crucial to better understand BTCs biology and therapeutic activity of molecularly targeted drugs.

## Abbreviations

ADC: antibody-drug conjugate
AE: adverse event
ALK: anaplastic lymphoma kinase
BRAF: v-RAF murine sarcoma viral oncogene homologue B1
BRCA: breast cancer gene
BTCs: biliary tract carcinomas
CAR: chimeric antigen receptor
CCA: cholangiocarcinoma
CEA: carcinoembryonic antigen
CI: confidence interval
Cis: cisplatin
DCR: disease control rate
DDR: DNA damage repair
DLT: dose-limiting toxicity
DoR: duration of response
eCCA: extrahepatic cholangiocarcinoma
EGFR: epidermal growth factor receptor
FDA: Food and Drug Administration
FGFR: fibroblast growth factor receptor
FISH: fluorescence in situ hybridization
GBC: gallbladder carcinoma
Gem: gemcitabine
GEMOX: gemcitabine plus oxaliplatin
HER: human epidermal growth factor receptor
HR: hazard ratio
iCCA: intrahepatic cholangiocarcinoma
IDH: isocitrate dehydrogenase
IHC: immunohistochemistry
IL: interleukin
IT: immunotherapy
KRAS: Kirsten rat sarcoma 2 viral oncogene homolog
MAPK: mitogen-activated protein kinases
MTD: maximum tolerated dose
NGS: next generation sequencing
ORR: objective response rate
OS: overall survival
PARP: poly ADP-ribose polymerase
PD-1: programmed cell death protein 1
PFS: progression-free survival
ROS1: c-ros oncogene1 receptor tyrosine kinase
RP2D: recommended phase 2 dose
VEGF: vascular endothelial growth factor

## Declarations

### Copyright

© The Author(s) 2021.
